# Type 1 Gastric Carcinoid in the Indian Population and Its Association with Multifocal Gastric Atrophy

**DOI:** 10.5005/jp-journals-10018-1180

**Published:** 2016-12-01

**Authors:** Anuradha Ananthamurthy, Marjorie Correa, Mallikarjun Patil

**Affiliations:** 1 Department of Pathology, St. John’s Medical College, Bengaluru, Karnataka, India; 2 Department of Gastroenterology, St. John’s Medical College, Bengaluru, Karnataka, India

**Keywords:** Atrophy, Carcinoid, Gastric, *H. pylori*, Neuroendocrine.

## Abstract

**Aim:**

Recent studies have shown an increase in the incidence of gastric neuroendocrine tumors (NETs) (carcinoids). This may be attributable to the frequent employment of endoscopy in clinical practice and the increasing use of proton pump inhibitors. From the literature that is available, it is interesting to note that the profile of patients with gastric carcinoids is different in the Asian population when compared to the western societies. As limited data is available from India, we evaluated retrospectively the clinical profile and pathology of gastric carcinoids presenting to our hospital.

**Materials and methods:**

A total of 31 patients with gastric carcinoids who presented to our institution from 2006 till 2013 were included in this study. The clinical data were obtained from the case files and the histopathology slides were reviewed.

**Results:**

Gastric carcinoids constituted about 32% of all gastrointestinal (GI) NETs and were second only to duodenal carcinoids in frequency. Men were more commonly affected (74%) and the majority were of type 1 (90%). Multifocal gastric atrophy with intestinal metaplasia was additional features seen in the majority of cases with type 1 carcinoids.

**Conclusion:**

This study, one of the largest series reported from India, shows that the frequency and profile of gastric carcinoids is different in this population when compared to the west. It also raises the possibility that *Helicobacter pylori* induced multifocal gastric atrophy might be a triggering factor for the most common type 1 gastric carcinoid rather than autoimmune gastritis.

**Clinical significance:**

Eradication of *H.* *pylori* may be a potential preventive strategy for the occurrence of gastric carcinoids.

**How to cite this article:**

Ananthamurthy A, Correa M, Patil M. Type 1 Gastric Carcinoid in the Indian Population and Its Association with Multifocal Gastric Atrophy. Euroasian J Hepato-Gastroenterol 2016;6(2):106-110.

## INTRODUCTION

Gastrointestinal (GI) neuroendocrine tumors (NETs) or gastric carcinoids constitute about 67.5% of all NETs.^1^ Small intestine, rectum, and appendix were thought to be common sites for occurrence of these tumors.^1,2^ Of late, the incidence of gastric and duodenal NETs have been reported to be increasing. The reasons for this increase may be several folds: The frequent use of upper GI endoscopy and the routine practice of obtaining biopsies, utilization of immunohistochemical techniques for diagnosis, and also the widespread use of proton pump inhibitors.

Gastric carcinoids represent distinct neoplasms that usually arise from the neuroendocrine cells of the stomach, namely the enterochromaffin-like (ECL) cells. The majority of these are discovered incidentally during endoscopy and do not cause any significant symptoms. However, a small percentage can show deep infiltration into the wall of the stomach as well as metastases.

Gastric NETs are endocrine tumors of the gastric mucosa and classified into three distinct types.^3^ Type 1 tumors are the commonest and are closely linked to chronic atrophic gastritis type A. Type 2 gastric NET is associated with Zollinger Ellison Syndrome (ZES) and occurs almost exclusively in the context of multiple endocrine neoplasia type 1 (MEN-1). Both these types are associated with hypergastrinemia. In type 1 gastric NET, hypergastrinemia is secondary to hypochlorhydria caused due to destruction of parietal cells. In type 2 NET, a primary gastrinoma induces ECL cell proliferation. In fact, hyperchlorhydria is a feature of type 2 gastric carcinoids. Type 1 NETs are located in the gastric fundus and body, may be multicentric, and are usually small. They tend to pursue a benign course and are believed to be more frequent in females in whom autoimmune gastritis is more common. Type 2 NETs also tend to be multiple and small and have low-grade malignant potential. They tend to occur in both males and females. Type 3 NET is not related to hypergastrinemia, is typically larger and more invasive, and may present with lymph node and distant metastases. Recently, a type 4 gastric NET has been described that arises from other endocrine cells, such as those producing serotonin or gastrin. These tumors are described to be very aggressive.

Most of the data pertaining to the epidemiology and clinical profile of GI carcinoids has emerged from western studies. There are very few studies of gastric NETs in the Indian literature. A few recent studies have indicated a rise in the incidence of gastric NETs even in the Indian population.^4^ We have noticed an increasing trend in the occurrence of gastric carcinoids in our setting, which is a Tertiary Care Center. In this study, we have examined the clinicopathological profile of gastric carcinoids over 8-year period, from 2006 till 2013. The associated morphological changes in the gastric mucosa were also noted with a special emphasis on patterns of atrophy.

## MATERIALS AND METHODS

A total of 31 patients diagnosed with gastric carcinoids from 2006 to 2013 were included in this study. The clinical data was obtained from the case files and the endoscopic findings were recorded. The histopathology slides were reviewed independently by two pathologists for the following tumor features: Size, presence of atypia, necrosis, mitotic count, and associated inflammatory cell response. The adjacent gastric mucosa was evaluated for atrophy and intestinal metaplasia.

## RESULTS

A total of 96 GI carcinoids were seen in the 8-year period in this institution. Of these, 30 were located in the stomach, 56 in the duodenum, and 1 patient had carcinoids in both the stomach and duodenum. Other uncommon sites were ileum, appendix, and rectum (Table 1). Among patients with gastric carcinoids, men were more commonly affected (n, 23). Anemia was one of the most common indications for performing an upper GI endoscopy (n, 15). Of the 31 gastric carcinoids, the majority (n, 28) were type 1 gastric carcinoids and 3 were of type 3. On esophago-gastro-duodenoscopy (OGD) examination, single tumors were seen in 13 patients and two or more tumors in 13. The tumors were classified as type 1 in 28 patients, whereas 3 patients had type 3 tumors. There were no patients with the type 2 tumors, which are usually associated with ZES. A majority of the tumors were located in the body and measured less than 1 cm (Table 2). Three of the type 1 tumors presented as polyps.

**Table 1:** **Table 1:** Distribution of carcinoids in the GI tract

*Site of carcinoid in the GI tract*		*Number of patients (n = 96)*	
Stomach		30 (31.5%)	
Duodenum		56 (58.5%)	
Stomach and duodenum		1 (1%)	
Ileum		4 (4%)	
Appendix		4 (4%)	
Colorectal		1 (1%)	

**Table 2:** **Table 2:** Clinical features and OGD findings of patients with gastric carcinoids

*Demographics and clinical features*		*Number (n = 31)*	
Age		31–75 years	
Sex		Male: 23 (74%), female: 8 (26%)	
*Symptoms/indication* for OGD			
Anemia		15	
Cholecystitis		2	
Pain abdomen		1	
Altered bowel habits		1	
Bleeding per rectum		1	
Acid peptic disease		1	
Not known		10	
*Number*/OGD findings			
Single		13	
More than one		13	
Normal mucosal study		1	
Not known		4	
*Location*: (OGD)			
Fundus		5	
Body		10	
Body and antrum		3	
Normal mucosal study		1	
Not known		12	
Size			
<1 cm		22	
1–2 cm		7	
>2 cm		2	

**Fig. 1:**
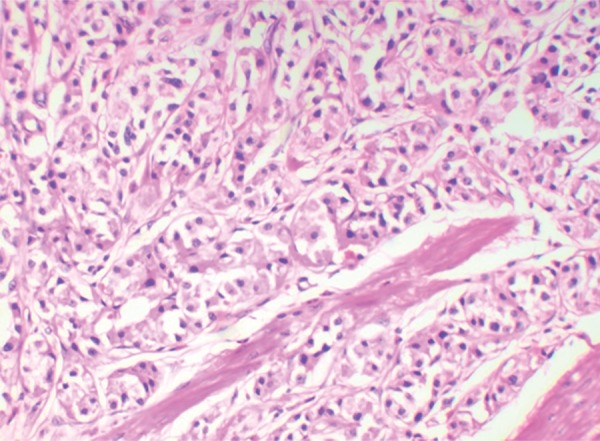
Gastric carcinoid showing the nesting pattern of cells. (H&E, 20×)

**Fig. 2:**
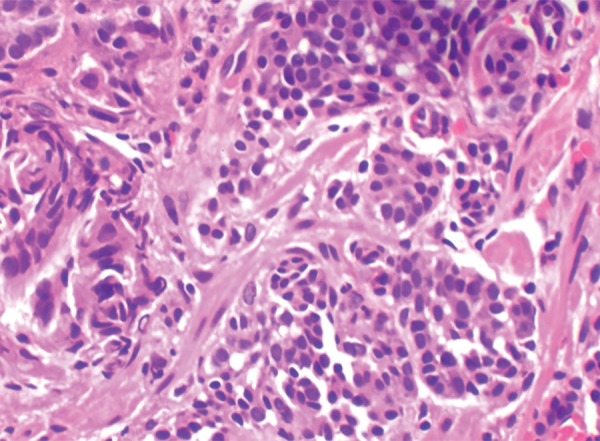
Gastric carcinoid showing the bland features of the nucleus. (H&E, 40×)

**Fig. 3:**
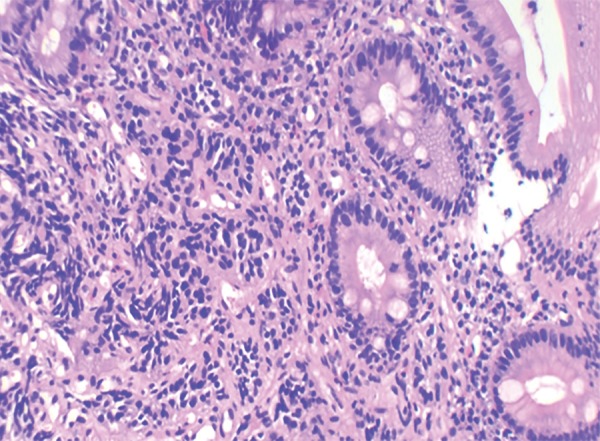
Gastric carcinoid with intestinal metaplasia in the adjacent epithelium. (H&E, 20×)

**Fig. 4:**
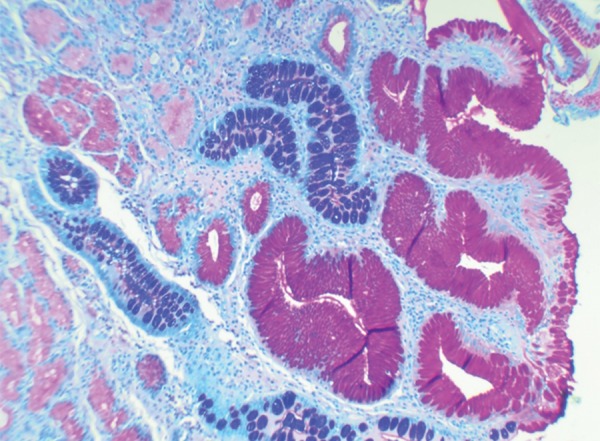
Intestinal metaplasia seen on AB PAS Stain (20×)

Histology showed all the tumors (100%) to be of low grade (G1). The cells showed a nesting pattern with bland nuclei and no atypia (Figs 1 and 2). No mitotic activity was seen in any of the tumors and there was no evidence of necrosis. A majority of the tumors also showed an infiltrate of eosinophils. Intestinal metaplasia in the adjacent mucosa as ratified by the Alcian Blue Periodic Acid–Schiff (AB-PAS) stain was seen in 20 cases of type 1 gastric carcinoids (Figs 3 and 4 and Table 3). The metaplastic changes were diffuse and were distributed both in the body and antral mucosal fragments. In three of the tumors, invasion of the muscularis propria was noted; and these three tumors also exhibited lymph node metastases. These tumors were all characterized as type 3 carcinoids. In patients with type 1 carcinoids, endoscopic removal was deemed adequate therapy, whereas the 3 patients with type 3 carcinoids underwent more extensive resections with lymph node dissections.

**Table 3:** **Table 3:** Histopathological findings of type 1 gastric carcinoids

*Histopathological findings*		*Number (n = 28)*	
*Atrophy*			
Atrophy with intestinal metaplasia		20	
No atrophy		5	
Atrophy cannot be assessed		3	
Grade			
G1		28	
G2		0	
G3		0	

## DISCUSSION

This study is a large series of gastric carcinoids seen in an Indian population. In a large series of 13,715 carcinoids from the USA, only 8.7% were of gastric origin.^1^ Other studies have shown a similar prevalence.^5^ A 50-year analysis of more than 500 gastric carcinoids showed definite increase in incidence in these tumors.^6^ In a recent Indian study of 74 GI NETs, stomach was the most commonly involved site (30.2%).^7^ This trend may due to the widespread application of upper GI endoscopy in patients with dyspepsia and unexplained anemia.^8^ The widespread use of proton pump inhibitors may also be a contributing factor. We have also seen an increasing incidence of gastric carcinoids in our institution, which are only second to duodenal carcinoids in frequency.

In this study, the majority of type 1 gastric NETs occurred in adult males, a feature unusual in the western population where type 1 carcinoids tend to occur in females in the background of autoimmune atrophic gastritis.^9^

In accordance to previous studies, type I carcinoids constituted a majority in our series.^9^ The tumors frequently occurred in the body and were multiple. A majority of type 1 carcinoids were less than 1 cm and were endoscopically excised. We had no cases of type 2 carcinoids, which occur in patients with MEN syndromes. Most studies show type 2 carcinoids to be rare. Type 3 carcinoids, also known as sporadic carcinoids, are known to be larger in size with an infiltrating growth pattern. All the three cases of sporadic carcinoids described in our study showed infiltration into the muscularis propria and also had lymph nodal metastases.

Varying degrees of metaplastic and nonmetaplastic atrophy were seen in the gastric mucosa in our patients with type 1 carcinoids. Gastric mucosal atrophy is believed to provide a background against which neuroendocrine proliferations occur. This is due to the increased levels of gastrin which stimulates ECL cells to proliferate. In western societies where autoimmune chronic atrophic gastritis is relatively commoner, type 1 NETs occur against this background.^10,11^ In India, where *Helicobacter pylori* infection is very common, multifocal atrophic gastritis caused by *H. pylori* is a more likely triggering factor in inducing gastric NETs. This is supported by the finding of multifocal atrophy in the antrum as well as fundus and body in our patients, whereas in autoimmune gastritis, atrophy is almost always restricted to the body. The finding of multifocal atrophy and also the higher prevalence of gastric NETs is suggestive of a *H.* *pylori-*related etiology in our population.

A study to differentiate intrinsic factor antibody (IFA) positive and IFA negative chronic atrophic gastritis among Indian patients concluded that they may be a spectrum of the same disease initiated by *H. pylori* infection.^12^ A few studies and reports have suggested that *H. pylori* infection induces formation of ECL cell carcinoids in the stomach.^13-15^

A majority of type 1 gastric carcinoids are less than 1 cm in greatest diameter and are cured by gastric endoscopic biopsies and polypectomies.^16^ More extensive surgery with lymph nodal dissections are warranted in tumors that are larger and more infiltrative, as seen in three of our cases which were type 3 carcinoids. These cases also exhibited lymph nodal metastases. Many studies have analyzed factors that predict outcomes in carcinoid tumors.^17,18^ However, these studies have not separately studied prognostic factors and outcomes in type 1 gastric carcinoids.

In conclusion, the increase in the incidence of type 1 gastric carcinoids coupled with the presence of multifocal gastric atrophy in these patients point toward a probable *H.* *pylori*-related pathogenesis. This is in contrast to the pathogenesis of type 1 gastric carcinoids occurring in the context of autoimmune gastritis in the western population. More studies are warranted to elucidate the actual mechanisms involved.
